# Supplementary material for: Developing and piloting an online course on osteoporosis using a multidisciplinary multi-institute approach- a cross-sectional qualitative study
Source: PLoS One. 2024 Feb 15;19(2):e0291617. doi: 10.1371/journal.pone.0291617 (PMC10868812; doi:10.1371/journal.pone.0291617)
Supplement: Attachment — Submitted filename: Reviewed PONE-D-22-28614_reviewer.pdf [file pone.0291617.s001.pdf]

PLOS ONE

# Developing and piloting an online course on osteoporosis using a multidisciplinary multi-institute approach- a cross-sectional qualitative study

--Manuscript Draft--

| | |
|---|---|
| Manuscript Number: | PONE-D-22-28614 |
| Article Type: | Research Article |
| Full Title: | Developing and piloting an online course on osteoporosis using a multidisciplinary multi-institute approach- a cross-sectional qualitative study |
| Short Title: | A multidisciplinary and multi-institute online course on bone disorders: Impact on teaching and learning |
| Corresponding Author: | Lena Jafri<br>Aga Khan University<br>Karachi, PAKISTAN |
| Keywords: | education; metabolic bone disease; e-learning; multidisciplinary collaboration; blended learning |
| Abstract: | Introduction<br><br>Postgraduates (PGs) trained via a multidisciplinary model receive collaborative learning opportunities from a wide range of experts. There is dearth of such approach in the current postgraduate medical education (PGME) curriculum in Pakistan. Taking the example of osteoporosis, a multidisciplinary online course on osteoporosis was designed to gauge the educational impact of a novel educational tool on teaching and learning capabilities of PGs and course facilitators.<br><br>Methods<br><br>A multi-institute multi-disciplinary team was formulated to deliver a course on osteoporosis on a virtual learning environment (VLE) platform. PGs (n=9) from various disciplines and institutes were nominated by their respective faculty and enrolled after taking informed consent. The modular component consisted of recorded micro-lectures, flash cards, video, case challenges, and mini-interview with the experts. Educational impact was measured by pre, and post module test and perception of the process was taken on a Likert scale of 1-5.<br><br>Results<br><br>Pre-test was attempted by 66% (n=6) PGs with a mean score of 43.8%. After taking the VLE module, all PGs (n=9) cleared the end of module test with an average score of 96%. PGs (n=9), 100%, believed that VLE was a great platform for online courses and were satisfied by this teaching strategy. They (n=8), 88.9%, stated that they could easily navigate the course and were comfortable using VLE. Majority (n=8), 88.9%, agreed that course learning was implementable in clinical practice, whereas 66.7% (n=6) were extremely satisfied with learning objectives and the content uploaded. The process also led to capacity building of facilitators to become acquainted with VLE.<br><br>Conclusion<br><br>An institute with adequate facilities for faculty training can develop online multidisciplinary modules to benefit institutes in resource limited settings. |
| Order of Authors: | Lena Jafri |
| | Hafsa Majid, FCPS Chemical Pathology |
| | Sibtain Ahmed |
| | Arsala Jameel Farooqui, BDS |
| | Muhammad Umer Naeem Effendi |
| | |

Powered by Editorial Manager® and ProduXion Manager® from Aries Systems Corporation

| | |
|---|---|
| | Qamar Riaz |
| | Noreen Nasir |
| | Sadia Fatima |
| | Sarah Nadeem |
| | Maseeh uz Zaman |
| | Rizwan Haroon Rashid |
| | Aamir Ejaz |
| | Nusrat Alvi |
| | Farheen Aslam |
| | Aysha Habib Khan |
| **Additional Information:** | |
| **Question** | **Response** |
| **Financial Disclosure**<br><br>Enter a financial disclosure statement that describes the sources of funding for the work included in this submission. Review the submission guidelines for detailed requirements. View published research articles from *PLOS ONE* for specific examples.<br><br>This statement is required for submission and **will appear in the published article** if the submission is accepted. Please make sure it is accurate.<br><br>**Unfunded studies**<br>Enter: *The author(s) received no specific funding for this work.*<br><br>**Funded studies**<br>Enter a statement with the following details:<br>• Initials of the authors who received each award<br>• Grant numbers awarded to each author<br>• The full name of each funder<br>• URL of each funder website<br>• Did the sponsors or funders play any role in the study design, data collection and analysis, decision to publish, or preparation of the manuscript?<br>• **NO** - Include this sentence at the end of your statement: *The funders had no role in study design, data collection and analysis, decision to publish, or preparation of the manuscript.*<br>• **YES** - Specify the role(s) played. | The authors received no specific funding for this work. |

| | |
|---|---|
| * typeset | |
| **Competing Interests**<br><br>Use the instructions below to enter a competing interest statement for this submission. On behalf of all authors, disclose any competing interests that could be perceived to bias this work—acknowledging all financial support and any other relevant financial or non-financial competing interests.<br><br>This statement is required for submission and **will appear in the published article** if the submission is accepted. Please make sure it is accurate and that any funding sources listed in your Funding Information later in the submission form are also declared in your Financial Disclosure statement.<br><br>View published research articles from *PLOS ONE* for specific examples.<br><br>**NO authors have competing interests**<br><br>Enter: *The authors have declared that no competing interests exist.*<br><br>**Authors with competing interests**<br><br>Enter competing interest details beginning with this statement:<br><br>*I have read the journal's policy and the authors of this manuscript have the following competing interests: [insert competing interests here]*<br><br>* typeset | The authors have declared that no competing interests exist. |
| **Ethics Statement**<br><br>Enter an ethics statement for this submission. This statement is required if the study involved:<br><br>• Human participants<br>• Human specimens or tissue | N/A |

Powered by Editorial Manager® and ProduXion Manager® from Aries Systems Corporation

| | |
|---|---|
| • Vertebrate animals or cephalopods<br>• Vertebrate embryos or tissues<br>• Field research<br><br>Write "N/A" if the submission does not require an ethics statement.<br><br>General guidance is provided below. Consult the submission guidelines for detailed instructions. **Make sure that all information entered here is included in the Methods section of the manuscript.** | |

*Powered by Editorial Manager® and ProduXion Manager® from Aries Systems Corporation*

| | |
|---|---|
| **Format for specific study types**<br><br>**Human Subject Research (involving human participants and/or tissue)**<br>• Give the name of the institutional review board or ethics committee that approved the study<br>• Include the approval number and/or a statement indicating approval of this research<br>• Indicate the form of consent obtained (written/oral) or the reason that consent was not obtained (e.g. the data were analyzed anonymously)<br><br>**Animal Research (involving vertebrate animals, embryos or tissues)**<br>• Provide the name of the Institutional Animal Care and Use Committee (IACUC) or other relevant ethics board that reviewed the study protocol, and indicate whether they approved this research or granted a formal waiver of ethical approval<br>• Include an approval number if one was obtained<br>• If the study involved *non-human primates*, add *additional details* about animal welfare and steps taken to ameliorate suffering<br>• If anesthesia, euthanasia, or any kind of animal sacrifice is part of the study, include briefly which substances and/or methods were applied<br><br>**Field Research**<br><br>Include the following details if this study involves the collection of plant, animal, or other materials from a natural setting:<br>• Field permit number<br>• Name of the institution or relevant body that granted permission | |
| **Data Availability**<br><br>Authors are required to make all data underlying the findings described fully available, without restriction, and from the time of publication. PLOS allows rare exceptions to address legal and ethical concerns. See the PLOS Data Policy and FAQ for detailed information. | No - some restrictions will apply |

Powered by Editorial Manager® and ProduXion Manager® from Aries Systems Corporation

| | |
|---|---|
| A Data Availability Statement describing where the data can be found is required at submission. Your answers to this question constitute the Data Availability Statement and **will be published in the article**, if accepted.<br><br>**Important:** Stating 'data available on request from the author' is not sufficient. If your data are only available upon request, select 'No' for the first question and explain your exceptional situation in the text box.<br><br>Do the authors confirm that all data underlying the findings described in their manuscript are fully available without restriction? | |
| **Describe where the data may be found in full sentences. If you are copying our sample text, replace any instances of XXX with the appropriate details.**<br><br>• If the data are **held or will be held in a public repository**, include URLs, accession numbers or DOIs. If this information will only be available after acceptance, indicate this by ticking the box below. For example: *All XXX files are available from the XXX database (accession number(s) XXX, XXX.*).<br>• If the data are all contained **within the manuscript and/or Supporting Information files**, enter the following: *All relevant data are within the manuscript and its Supporting Information files.*<br>• If neither of these applies but you are able to provide **details of access elsewhere**, with or without limitations, please do so. For example:<br><br>*Data cannot be shared publicly because of [XXX]. Data are available from the XXX Institutional Data Access / Ethics Committee (contact via XXX) for researchers who meet the criteria for access to confidential data.*<br><br>*The data underlying the results presented in the study are available from (include the name of the third party* | The authors have declared that no competing interests exist. |

| | |
|---|---|
| *and contact information or URL).*<br>• This text is appropriate if the data are owned by a third party and authors do not have permission to share the data.<br><br>* typeset | |
| Additional data availability information: | |

Manuscript
Click here to access/download;Manuscript;MANUSCRIPT_MOOC_Qualitative

# Developing and piloting an online course on osteoporosis using a multidisciplinary multi-institute approach- a cross-sectional qualitative study

Lena Jafri*[1], Hafsa Majid[1], Sibtain Ahmed[1], Arsala Jameel Farooqui[1], Muhammad Umer Naeem Effendi[1], Maseeh-uz-Zaman[2], Qamar Riaz[3], Noreen Nasir[4], Sadia Fatima[5], Sarah Nadeem[4], Rizwan Haroon Rashid[6], Aamir Ejaz[7], Nusrat Alvi[8], Farheen Aslam[9], Aysha Habib Khan[1]

1. Section of Chemical Pathology, Department of Pathology and Laboratory Medicine, Aga Khan University, Karachi, Pakistan
2. Department of Radiology, Aga Khan University, Karachi, Pakistan
3. Department of Education Aga Khan University, Karachi, Pakistan
4. Department of Medicine, Aga Khan University, Karachi, Pakistan
5. Department of Biological & Biomedical Sciences Aga Khan University, Karachi, Pakistan
6. Department of Surgery, Aga Khan University, Karachi, Pakistan
7. Mohi Uddin Islamic Medical College, Mirpur Azad Jamu and Kashmir, Pakistan
8. Rahbar Medical and Dental College, Lahore, Pakistan
9. Quaid e Azam Medical College, Bahawalpur, Pakistan

*Corresponding Author

Lena Jafri

lena.jafri@aku.edu

# Abstract

## Introduction

Postgraduates [illegible]Gs) trained via a multidisciplinary model receive collaborative learning opportunities from a wide range of experts. There is dearth of such approach in the current postgraduate medical education (PGME) curriculum in Pakistan. Taking the example of osteoporosis, a multidisciplinary online course on osteoporosis was designed to gauge the educational impact of a novel educational tool on teaching and learning capabilities of PGs and course facilitators.

## Methods

A multi-institute multi-disciplinary team was formulated to deliver a course on osteoporosis on a virtual learning environment (VLE) platform. PGs (n=9) from various disciplines and institutes were nominated by their respective faculty and enrolled after taking informed consent. The modular component consisted of recorded micro-lectures, flash cards, video, case challenges, and mini-interview with the experts. Educational impact was measured by pre, and post module test and perception of the process was taken on a Likert scale of 1-5.

## Results

Pre-test was attempted by 66% (n=6) PGs with a mean score of 43.8%. After taking the VLE module, all PGs (n=9) cleared the end of module test with an average score of 96%. [illegible] (n=9), 100%, believed that VLE was a great platform for online courses and were satisfied by this teaching strategy. They (n=8), 88.9%, stated that they could easily navigate the course and were comfortable using VLE. Majority (n=8), 88.9%, agreed that course learning was implementable in

clinical practice, whereas 66.7% (n=6) were extremely satisfied with learning objectives and the content uploaded. The process also led to capacity building of facilitators to become acquainted with VLE.

## Conclusion

An institute with adequate facilities for faculty training can develop online multidisciplinary modules to benefit institutes in resource limited settings.


## Keywords

education, metabolic bone disease, e-learning, multidisciplinary collaboration, blended learning


# Introduction

With a growing population in Pakistan (currently 217 million), the need for experts in the field also increases[illegible]fe expectancy in Pakistan has expanded from 46.6 years in 1960 to 65.4 years in 2010 and is anticipated to arrive at 72.4 years in 2023 [1]. Population aging and rising rates of osteoporosis, vitamin D deficiency, decreased calcium intake and sedentary lifestyles in Pakistan are some of the rising concerns related to bone health [2-5]. It is evaluated that the expense of hip fractures in Pakistan ranges between 4000 to 10000 USD relying upon the hospital setting with an average hospital remain of 7-10 days [6, 7].

A large majority of the educational structures in Pakistan rely on face-to-face teaching. Conventional education pattern of face-to-face teaching or organized projects using printed media cannot meet the adapting needs of healthcare professionals working over a various range of healthcare settings. The utilization of web-based learning has developed over the previous decade

and offers an adaptable and cost-productive technique to provide education to enormous amounts of learners [8, 9].

Taking metabolic bone disorders as an example for this online course, we will [illegible] able to reach postgraduate students living in remote areas and smaller cities, giving them the opportunity to access valuable course material. The core elements of this course will serve to enhance critical thinking skills, educate about bone diseases and to apply standard practices across Pakistan. This in turn will standardize care of patients suffering from bone diseases across Pakistan leading to correct and timely diagnosis and management and better outcomes. Teaching via virtual learning environment (VLE) can then be replicated for creating courses in other sub-specialties.

Through this pilot we aimed to highlight the efforts put in to develop and execute an online course, its educational impact and the evaluation provided by participants and facilitators.

# Materials and Methods

A descriptive multi methods cross-sectional study was conducted in the section of Chemical Pathology, Department of Pathology and Laboratory Medicine, Aga Khan University (AKU), Karachi, Pakistan. The study was conducted from January 2018 to December 2020. The Aga Khan University's ethical review committee's acceptance (ERC number 5415-Pat-ERC-18) was taken, and faculty and PGs provided informed consent before the pilot was conducted.

The course director created a multidisciplinary facilitator team that included faculty from various institutes. The College of Physicians and Surgeons Pakistan's (CPSP) registered postgraduate residents [illegible]G) from various specialties were invited to participate. To make sure the results of each stage meet their mission, and that the course module team is ready to smoothly transition to the next phase, we developed the workflow in following five phases, outlined in Fig 1.

**Fig 1: Methodological Workflow of the Study**

## Phase I – Need assessment via focus group discussions

The project team felt the need to have the PGs' perception of an online course on osteoporosis before developing the course content. Before initiating the discussion, written informed consent was taken from each PG trainee. Consent was taken via emails from PGs residing in other cities and it was clearly communicated that they were being invited to participate in this study as learners only and had the right to refuse. They were informed that there were no right or wrong answers to the questions to be asked and were expected to have differing points of view. The entire process of FGDs is summarized in Fig 2.

**Fig 2: Flowchart Outlining the process of Focus Group Discussions**

## Phase II –Faculty development

A multi-disciplinary, multi-institute facilitator team was formulated with people who shared a common interest in designing an online course on MBD. [illegible]complete hands-on workshop was conducted on Moodle by the blended and digital learning network of the institute with facilitators from other cities connected via online platform Zoom.

## Phase III – Developing course content

The course blueprint and course content were designed keeping in view the needs of PG trainees and challenges highlighted in previously conducted FGDs. The team tried minimizing required reading and relevant questions were developed to assess the trainees. The faculty developed course outlines to relate the pathophysiology of different metabolic bone diseases (MBDs) with risk factors and signs and symptoms. Special focus was laid on teaching participants how to order appropriate investigations to make a diagnosis of MBDs and prescribe pharmacologic and non-pharmacological interventions for the prevention and treatment of MBDs.

The course content included a series of required readings, lectures, videos, flashcards, case challenges, and discussion forums. Screengrabs of the VLE course platform are shown in Figs 3a, 3b and 3c.

**Fig 3a: Example of Pre-course Readings Required for Undertaking the VLE Course**

**Fig 3b: Example of Diagnostic Case Challenge Included in the VLE Course**

**Fig 3c: Risk Factors Associated with the Diagnostic Case Challenge**

## Phase IV – Course execution

After blueprinting Moodle with the course outline, the facilitators identified PGs from different disciplines and various institutes across Pakistan, who were then enrolled on Moodle to take up the course at their convenience. Email communications and e-meetings were carried out to keep team updated and active team members were acknowledged timely. Announcements were made on the VLE platform every time an open discussion on a particular topic was planned. A MOOC WhatsApp group was also created to keep participants and active team members in the loop and address the PGs' issues when required.

## Phase V – Course evaluation and feedback

Before the required reading for the module and other education material were made available, PGs were made to take the pre-module test which consisted of scenario-based multiple-choice questions. After the module was completed, the participants had to appear in the end-of-module quiz to gauge the gain in their knowledge. The post-test consisted of eight MCQs covering the learning objectives of the osteoporosis module. The levels were set as follows:

- Pretest
- Formative: EMQ, cases. Feedback will be given to those below the level.
- Summative (to determine terminal performance) or post-test:

- Selection of type of test paper: Best Choice Questions, Real world problems or cases will be used. Level of difficulty in Best Choice Questions and Extended Matching Questions will be C2 and C3 only.
- Each module and end of course summative exam need to be passed. Passing score would be 65%.
- Those who fail will be given some time to revisit the teaching material and re-attempt the quiz until they pass. (time lapse, bank, 1/2 supplementary attempts allowed)

Besides this, an online survey was performed to gather feedback from PGs and facilitators regarding their experience of the pilot MOOC in terms of course content and satisfaction of this innovative teaching and learning method on a Likert scale of 1-5.

# Statistical Analysis

The qualitative analytical method proposed by Braun and Clarke[illegible]s used to conduct thematic analysis of the FGD following the six phases. The six steps of analysis included data familiarization, generating initial codes, identifying themes, reviewing themes, defining themes, and report writing. Moderators played back the tapes, prepared and edited typescripts, performed coding, and systemically classified the information. Data collected was checked for accuracy by the principal investigator, and all team members reviewed the FGD report.

For quantitative data of FGDs, and PGs and facilitators perspective about the course, data was analyzed by Microsoft Excel. Frequency and percentages were generated.

# Results

## Phase I – need assessment via focus group discussions

In total, two FGDs were conducted which lasted for one and a half hours to two hours each. Each FGD consisted of 5-7 participants. Altogether fourteen PG trainees participated in the two FGDs,

who were from all levels of residency training (Years 1-6). The male to female ratio was 2:1. Residents from AKU-Karachi took part in the discussion physically, while residents from Rehman Medical Centre-Peshawar, and Quaid-e-Azam University-Bahawalpur, participated remotely via Zoom. Four main themes emerged from analysis of PGs discussions in FGDs: need of course, time commitments, tools and technology and course content/assessment, which are summarized in Table 1.

**Table 1: Results of the Focus Group Discussion with Postgraduate Residents (n=14) on Needs of Metabolic Bone Disorders Online Course in their Training:**

| Themes | Findings of Focus Group Discussion -PGs responses | n -(%) |
|---|---|---|
| **The need for Metabolic Bone Disorders -course on Virtual Learning Environment** | There is an urgent need for a course on Metabolic Bone Disorders | 14 (100) |
| | The burden of Metabolic Bone Disorders is high in Pakistan | 8 (57.1) |
| | Current residency training does not appropriately cover objectives related to bone disorders | 10 (71.4) |
| **Time Commitment** | Weekdays-early evenings are the preferred time to access the Metabolic Bone Disorders course | 9 (64.2) |
| | Total time that can be taken out from residents' schedule is 1-2 hours per week | 9 (64.2) |
| **Tools and Technology use** | Course access via laptop is preferred over cellphone | 9 (64.2) |
| | Comfortable in using social media group as a tool for connecting with course faculty | 12 (85.7) |
| | Internet connectivity and bandwidth may become a barrier in course access | 10 (71.4) |
| **Course content and assessment** | Such courses on VLE should include short or micro lectures and/or downloadable presentations | 12 (85.7) |

| | | |
|---|---|---|
| | Best choice questions should be used for end of module assessments | 14 (100) |

## Phase II – faculty development

A multi-disciplinary, multi-institute facilitator team (n=17) was formulated with experts from Radiology, Orthopedics, Medicine, Endocrinology, Chemical Pathology and Education, from across Pakistan.

The institution's blended and digital learning network trained the facilitators (n=7) on how to adapt themselves to virtual learning environment (Moodle). Research assistant and associates from the department of Pathology and Laboratory Medicine, AKU, were also trained in this workshop. The entire process led to development and strengthening of skills, instinct, and abilities that organizations and communities need to survive, adapt, and thrive in a fast-changing world.

## Phase III – developing course content

The team developed a blueprint for the four modules of the course. Four modules were decided: osteoporosis, osteomalacia and rickets, parathyroid disorders, and rare bone diseases. Osteoporosis was chosen as the pilot module and its complete course content was uploaded on Moodle, outlined in Tables 2a and 2b.

**Table 2a: Material developed for Osteoporosis Module on Virtual Learning Environment by Multidisciplinary Facilitator Team**

| Type of content | Description | Contact Time |
|---|---|---|
| **Flash cards** | - Bone turnover markers<br>- FRAX limitations<br>- Fracture Liaison Services | - 10 mins<br>- 10 mins<br>- 10 mins |

| | | |
|---|---|---|
| **Videos** | - Overview of Osteoporosis<br>- Secondary Osteoporosis<br>- DEXA-scan procedure and interpretation<br>- Expert's insight on Osteoporosis treatment | - 8 mins<br>- 10 mins<br>- 6 mins 30 secs<br>- 4 mins 15 secs |
| **Documents** | - Course curriculum<br>- Overall learning outcomes<br>- Learning objectives of module 1<br>- Faculty profiles | - 5 mins<br>- 5 mins<br>- 10 mins |
| **Educational content** | - Pre-course reading on bone structure and remodeling by senior faculty<br>- Review article | - 10 mins<br>- 20 mins |
| **Pre-test, end of module quiz and Case challenges** | - MCQs based pre-test and end of module quiz<br>- Scenario based case challenges on osteoporosis pathophysiology, diagnosis and management | - 40 mins<br>- 45 mins |

*Abbreviations: FRAX (fracture risk assessment score), mins (minutes), secs (seconds), MCQs (multiple choice questions)*

**Table 2b: Learning Objectives for the Osteoporosis Module**

| **Course Objectives** |
|---|
| • Understand the prevalence of osteoporosis in by age and gender in Pakistan |
| • Define fragility fractures and its burden in Pakistan |
| • Identify most common and least likely sites of fragility fractures |
| • Diagnose fragility fractures based on relevant biochemical investigations |
| • Suggest measures to reduce recurrent fractures |
| • Understand the role of nutrition and lifestyle factors in prevention of osteoporosis |
| • Recommend dietary calcium intake for patients with osteoporosis |
| • Know the risk factors included in FRAX |
| • Apply fracture risk assessment tool on patients |
| • Appreciate the limitations of FRAX in Pakistani population |
| • List different bone turnover markers and describe its clinical utility and limitations |
| • List indication for performing X-rays, DXA scans in patients with osteoporosis |
| • Order appropriate and cost-effective investigations including X-rays, DXA scans (ensuring differentiation Z-score and T-score) |

| | |
|---|---|
| | • Interpretation DXA and make a diagnosis of osteoporosis and osteopenia based on DXA report |
| | • List the indications for ordering a vertebral fracture assessment (VFA) and the limitations of VFA |
| | • Knowledge of other available methods for BMD measurement |
| | • Know the mechanism of action, use and rare side effects of following drugs:<br>o Oral bisphosphonates – alendronate, risedronate and ibandronate<br>o IV bisphosphonates – zoledronate, ibandronic acid<br>o Strontium ranelate<br>o Denosumab<br>o Raloxifene<br>o Teriparatide<br>o HRT (with/without progesterone)<br>o Calcium<br>o Vitamin D |
| | • Prescribe pharmacologic and non-pharmacological interventions for the prevention and treatment of osteoporosis |
| | • Understand role of a multidisciplinary group in management of osteoporotic fracture |
| | • List the various components in a FLS |
| | • Develop liaison for FLS and the pathway for its implementation |

The faculty developed the content based mostly on case scenarios posing critical thinking and decision making. The teaching tools used as the course guide for the facilitators are shown in Table 3. Challenges and fears of PGs from FGDs were critically analysed by the project team and facilitators, who then produced solutions to the problems, which are summarized in Table 4. Questions developed for assessment at the end of the module were reviewed by educational experts and content related evidence was reviewed by five other facilitators for validity and feasibility.

**Table 3: Results of Focus Group Discussion with Metabolic Bone Disorders Online Course Facilitators on its Designing and Execution**

| Challenges identified | Solutions proposed |
|---|---|
| Need for e-course | Pedagogical model prepared |
| Short attention spans of PG trainees | Prepared Micro lectures (max 7 min) |
| Interactive Course Content | Teaching and learning activities in the form of case challenges and discussion forum |
| Internet connectivity issues | Downloadable lectures |
| Busy schedule of trainees | Course access flexibility |
| Limited learning time available to trainees | Kept it asynchronous with division into multiple modules |

**Table 4: Teaching tools Used for the Osteoporosis Module**

| Days | Activity | Accessibility | Student engagement level | Faculty engagement level during the course | Contact Time in min |
|---|---|---|---|---|---|
| **3 days before opening the course** | Open Welcome Message by Course Director<br>Open Course Outline<br>Ice breaking Activity | Open till end<br>Open till end Open till end | +<br>+<br>+++ | -<br>-<br>+++ | 5<br>5<br>15 |
| **1** | Open Module 1 objectives<br>Open Required Reading for module 1<br>Open Video Lecture on Osteoporosis | Open till end<br>Open till end<br>Open till end | +<br>+<br>++ | -<br>-<br>+ | 10<br>20<br>8 |
| **2** | Case Challenge 1 | Open till end | +++ | - | 15 |
| **3** | Open Video on Sec Osteoporosis<br>Open flash card on bone turnover markers | Open till end<br>Open till end | ++<br>+ | +<br>- | 10<br>10 |
| **4** | Case Challenge 2 | Open till end | +++ | - | 15 |
| **5** | Open both Videos on DEXA Scan | Open till end | +++ | - | 5:30<br>1 |
| **6** | Case Challenge 3 | Open till end | +++ | - | 15 |
| **7** | Open Discussion Forum on FRAX | Open for 4 days | +++ | +++ | 15 |
| **8** | Open Flash Card on FRAX limitations | Open till end | + | - | 10 |
| **9** | Case Challenge 4 | Open till end | +++ | - | 30 |
| **10** | Open video on experts' insight into osteoporosis treatment | Open till end | +++ | - | 4:15 |
| **11** | Buffer Time | - | - | - | |
| **12** | Open Flash card on Fracture Liaison Service | Open till end | + | - | 10 |
| **13** | Buffer Time | - | - | - | |
| **14** | End of module test | Open for 2 days | +++ | - | 20 |
| **16** | Course completed; VLE shell hidden from students; certificate provided to those who attempted end of module quiz | Open for 5 days | + | +++ | |

*Students contact time: 209 minutes in all =3.4 hours min to 5 hours max*

## Phase IV – course execution

The pilot for the osteoporosis module was spread over a duration of one month (June–July 2019). Ten PG trainees were enrolled in the course. These PGs included fellows and residents' years 1-5 from Chemical Pathology (n=6), Endocrinology (n=1), Radiology (n=1) and Internal Medicine (n=1) belonging to institutes of Aga Khan University Karachi, Chughtai Institute of Pathology, Lahore, Rehman Medical Institute, Peshawar and Quaid-e-Azam Medical College, Bahawalpur. The PGs attempted the pre-test and completed the osteoporosis module. Three attempts were allowed for the end of module assessment, two trainees performed it thrice while the rest attempted it twice. In end of module assessment eight participants scored 100% while one each scored 87.5% and 50%.

## Phase V – course evaluation and feedback

Results of the pre-test and end of module quiz are shown in Fig 4. Feedback of PGs on the course was based on their experience of the VLE platform as a source of teaching and learning, and course content delivered. Most of the participants were in strong consensus for this way of teaching and learning and were satisfied with the course content as shown in Fig 5a. Around 47% (8/17) of the facilitators participated in an online survey to provide their feedback of virtual teaching experience. All participants were extremely contented with various aspects of this innovative teaching methodology; however, they shared that they still need further assistance to master the art (Fig 5b).

**Fig 4: Pre and Post-test Scores for the Osteoporosis Module of Postgraduate trainees.** The dark horizontal line represents the passing score (>75%)

**Fig 5a: Feedback of Postgraduate trainees for VLE as a teaching-learning methodology and Course Content (n=9)**

**Fig 5b: Feedback of Course Facilitators for VLE as a teaching-learning methodology and Course Content (n=8)**

# Discussion

E-learning has shown to be one of the tools that addresses some of the training challenges experienced in developing countries by supporting efficient content delivery, decreasing costs, and increasing access [10]. Qualitative analysis of the participating PGs' perspectives in FGDs revealed themes that helped design a completely student-focused online course for osteoporosis. Current gaps in course content were outlined by the PGs who shared that they were not taught sufficient material about MBDs, and the course content was consequently developed to address topics that would be relevant during their residency exit examinations. According to the trainees, the average time that they would devote to studying the BMD course was 2 hours per week, hence the duration of the course content was customized accordingly. Moreover, most of them wanted to learn in the afternoon during the weekdays on-campus. A discovery by Green et al. (2014) also showed that students on blended courses preferred using on-campus time more, primarily to discuss with other colleagues [11].

With the advent of innovative educational methodologies such as flipped classrooms, live online lectures are being utilized for distant learning programs. However, our focus group had a contrasting opinion, with only two participants in concordance with the idea of using this approach. The group claimed that live lectures require a dedicated time slot, which is challenging to manage with routine duties. Majority of participants were in favor of short downloadable presentations or micro-lectures, which have comprehensive content and would enable PGs to study as per their convenience.

Continuity in learning as an essential aspect of online learning has positively shown to affect overall gain in skills and knowledge in clinical reasoning [12]. The participating PGs also

positively responded to the VLE platform, appreciating that it removed their digital inexperience and providing them with a flexible remote learning strategy. Numerous studies performed through questionnaires also showed high acceptance of using computer assisted learning [13, 14]. The findings of this study strengthened the results of previously evaluated studies where students preferred using computer-based educational material [15-17]. It must be acknowledged that although the VLE platform for this course is designed to be user-friendly and is accessible by computers and handheld devices, i.e., smartphones and tablets, some PGs shared concerns about internet connectivity problems being a possible barrier to access.

E- assessment and evaluation of practice are part of the e- learning strategy and are thus essential to ensure its utility. On being asked what point of the course evaluations should be carried out at, the cluster had a diversified opinion, with most in favour of end of module evaluations. This methodology is evidenced to make participants more focused to qualify for the next session, with the provision of scores in each module nurturing a competitive learning environment [18]. On a bigger scale, E-assessment also permits institutions to widen the range of evaluation practices and improves the impartiality and stability of their assessment. It also diminishes the marking burdens and helps the organization and assessment programs and evaluation techniques [19].

The group had a consensus regarding the use of BCQs as the mode of assessment. They accentuated that the focus of BCQs should be on diagnostic, case based, data driven approaches to feed their diagnostic capabilities of MBDs. In their view, case-based teaching and assessment provide better judgment of a candidate’s understanding of the entire clinical spectrum.

Findings of our study support the importance of faculty development by designing an online course that is effective, flexible, and accessible, after overcoming all challenges and hurdles. Upgrading the aptitudes of faculty members as postgraduate supervisors is imperative to ensure a fruitful

supervision experience and to diminish the whittling rates among PG students [20]. On-going research reveals that MOOCs offer a favorable solution for faculty development [21]; associated advantages of utilizing a MOOC with faculty peers may incorporate expanded awareness and comprehension about web-based learning through direct insight.

In this pilot course on osteoporosis, having faculty from multiple disciplines including Radiology, Orthopedics, Medicine, Endocrinology, Chemical Pathology and Education strengthened the quality of course material delivered to the PGs. Prospectively, the course may include faculty from Dentistry, as studies highlight the significance of full-mouth X-rays or orthopantomograms (OPGs) in establishing a diagnosis of osteoporosis. According to recent studies, radiographic findings on OPGs can help the dentist identify patients with low bone density, and then refer them to specialists for BMD assessment [22]· Computer active shape modelling technology can be used to detect bone density on OPGs by measuring the cortical width of the bone on the lower border of the mandible. This automatic method is reproducible and has good diagnostic accuracy (Az = 0·759 (95% CI = 0·724–0·791) [23]. This finding is supported by the meta-analysis performed by Calciolari et al, which explored three different mandibular bone measurement indices with variable sensitivity and reported the best specificity in measuring the mandibular cortical width, also known as the mandibular cortical thickness, or mental index [24]. Given its abundant citations in literature, the utility of dental OPGs in detecting osteoporotic risk is an area that can be inexpensively explored and incorporated in future curricular courses.

If online curricula are constructed and organised diligently, students across different institutions can easily access open academic lectures at ease. If PGs are specifically targeted and course content is related to their professional needs and research interests, online learning platforms can prove to be greatly beneficial to their academic growth and success [25]. The present study conducted on

postgraduate medical education curriculum needs was the first of its kind in Pakistan. The type of study design gave a clearer picture of various PGs' perceptions towards the acceptance of distance learning. Their perspectives helped the project team in designing a successful MBD online course catering to the exposed challenges.

# Conclusion and Recommendation

In this digital era, it is imperative that PGs are exposed to learning pedagogical strategies via the virtual learning environment. The VLE platform has proved to provide PGs with a supportive learning tool that addresses training challenges faced by developing countries, including cost, content delivery and remote accessibility. Findings of this study will be essential for faculty members, institutions' administrations and e-learning developers who are keen to effectively design MOOCs as a pliable and constructive source for learning.


## Acknowledgments

We want to acknowledge Nadir Shah, Department of IT Academics and Computing, AKUH for his support throughout this project.

# References


1. Jalal S, Younis MZ. Aging and elderly in Pakistan. Ageing International. 2014 Mar;39(1):4-12.
2. Khan AH, Iqbal R, Naureen G, Dar FJ, Ahmed FN. Prevalence of vitamin D deficiency and its correlates: results of a community-based study conducted in Karachi, Pakistan. Archives of osteoporosis. 2012 Dec;7(1):275-82.
3. Iqbal R, Khan AH. Possible causes of vitamin D deficiency (VDD) in Pakistani population residing in Pakistan. J Pak Med Assoc. 2010 Jan 1;60(1):1-2.
4. Dar FJ, Iqbal R, Ghani F, Siddiqui I, Khan AH. Bone health status of premenopausal healthy adult females in Pakistani females. Archives of osteoporosis. 2012 Dec;7(1):93-9.
5. Lowe NM, Ellahi B, Bano Q, Bangash SA, Mitra SR, Zaman M. Dietary calcium intake, vitamin D status, and bone health in postmenopausal women in rural Pakistan. Journal of health, population, and nutrition. 2011 Oct;29(5):465.
6. Baig L, Mansuri FA, Karim SA. Association of menopause with osteopenia and osteoporosis: results from population based study done in Karachi. J Coll Physicians Surg Pak. 2009 Apr 1;19(4):240-4.
7. Handa R, Kalla AA, Maalouf G. Osteoporosis in developing countries. Best practice & research Clinical rheumatology. 2008 Aug 1;22(4):693-708.
8. George PP, Papachristou N, Belisario JM, Wang W, Wark PA, Cotic Z, Rasmussen K, Sluiter R, Riboli–Sasco E, Car LT, Musulanov EM. Online eLearning for undergraduates in health professions: a systematic review of the impact on knowledge, skills, attitudes and satisfaction. Journal of global health. 2014 Jun;4(1).

**9.** Rocha-Pereira N, Lafferty N, Nathwani D. Educating healthcare professionals in antimicrobial stewardship: can online-learning solutions help?. Journal of Antimicrobial Chemotherapy. 2015 Dec 1;70(12):3175-7.

**10.** Ertmer PA, Nour AY. Teaching basic medical sciences at a distance: strategies for effective teaching and learning in internet-based courses. Journal of veterinary medical education. 2007 Jul;34(3):316-24.

**11.** Green RA, Farchione D, Hughes DL, Chan SP. Participation in asynchronous online discussion forums does improve student learning of gross anatomy. Anatomical sciences education. 2014 Jan;7(1):71-6.

**12.** Salim H, Lee PY, Ghazali SS, Ching SM, Ali H, Shamsuddin NH, Mawardi M, Kassim PS, Dzulkarnain DH. Perceptions toward a pilot project on blended learning in Malaysian family medicine postgraduate training: a qualitative study. BMC medical education. 2018 Dec;18(1):1-0.

**13.** Garrett TJ, Ashford AR, Savage DG. A comparison of computer-assisted instruction and tutorials in hematology and oncology. Journal of Medical Education. 1987 Nov 1;62(11):918-22.

**14.** Schmulian C, Kenny GN, Campbell D. Use of microcomputers for self-assessment and continuing education in anaesthesia. Br Med J (Clin Res Ed). 1982 Feb 6;284(6313):403-5.

**15.** Cheng A, Lang TR, Starr SR, Pusic M, Cook DA. Technology-enhanced simulation and pediatric education: a meta-analysis. Pediatrics. 2014 May;133(5):e1313-23.

**16.** Handler TJ, Lynch P, Jaffe CC. Computer-aided learning validation: a CAI-critical mission. InProceedings of the Annual Symposium on Computer Application in Medical Care 1995 (p. 522). American Medical Informatics Association.

**17.** Plasschaert AJ, Wilson NH, Cailleteau JG, Verdonschot EH. Opinions and experiences of dental students and faculty concerning computer-assisted learning.

**18.** Sidebotham M, Baird K, Walters C, Gamble J. Preparing student midwives for professional practice: Evaluation of a student e-portfolio assessment item. Nurse Education in Practice. 2018 Sep 1;32:84-9.

***19.*** Beggs, K. and Cantillon, P. (2019) E-assessment. *FAIMER-Keele Master's in Health Professions Education: Accreditation and Assessment*. Module 3, Unit 2. FAIMER Centre for Distance Learning, CenMEDIC, London.

**20.** Erichsen EA, Bolliger DU, Halupa C. Student satisfaction with graduate supervision in doctoral programs primarily delivered in distance education settings. Studies in Higher education. 2014 Feb 7;39(2):321-38.

**21.** Bond P. Massive Open Online Courses (MOOCs) for professional development and growth. InContinuing education for librarians: essays on career improvement through classes, workshops, conferences and more 2013 Jan 30 (p. 28). McFarland.

**22.** Parlani S, Nair P, Agrawal S, Chitumalla R, Beohar G, Katar U. Role of panoramic radiographs in the detection of osteoporosis. Journal of Oral Hygiene & Health. 2014 Feb 28:2-4.

**23.** Devlin H, Horner K. Diagnosis of osteoporosis in oral health care. Journal of oral rehabilitation. 2008 Feb;35(2):152-7.

**24.** Calciolari E, Donos N, Park JC, Petrie A, Mardas N. Panoramic measures for oral bone mass in detecting osteoporosis: a systematic review and meta-analysis. Journal of dental research. 2015 Mar;94(3_suppl):17S-27S.

**25.** Li Y, Wen X, Li L, Zhou YE, Huang L, Ling B, Liao X, Tang Q. Exploration of Online Education Mode for Postgraduate Education under the Background of COVID-19. Advances in Applied Sociology. 2021 May 7;11(05):223

Figure

Click here to access/download;Figure;MOOC Figures_FINAL.docx

**Fig 1**

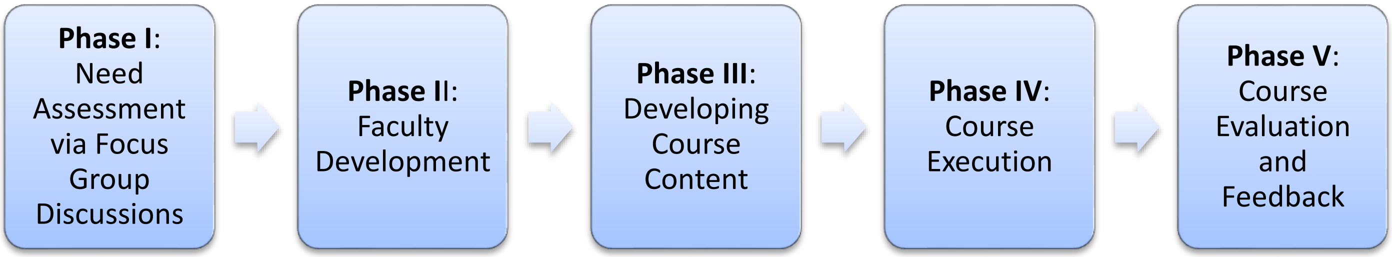

**Fig 2**

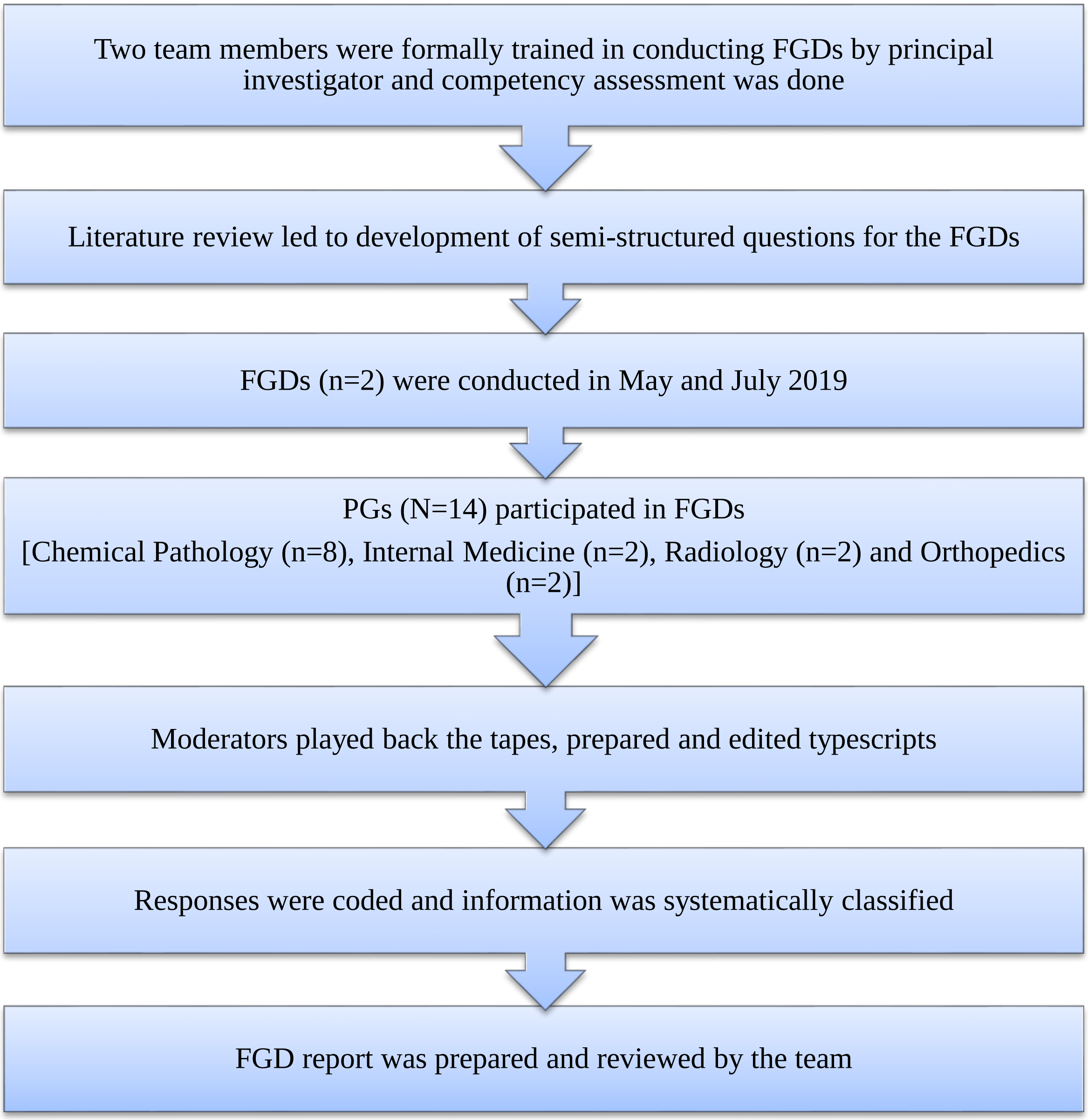
Two team members were formally trained in conducting FGDs by principal investigator and competency assessment was done
Literature review led to development of semi-structured questions for the FGDs
FGDs (n=2) were conducted in May and July 2019
PGs (N=14) participated in FGDs
[Chemical Pathology (n=8), Internal Medicine (n=2), Radiology (n=2) and Orthopedics (n=2)]
Moderators played back the tapes, prepared and edited typescripts
Responses were coded and information was systematically classified
FGD report was prepared and reviewed by the team

## Fig 3a

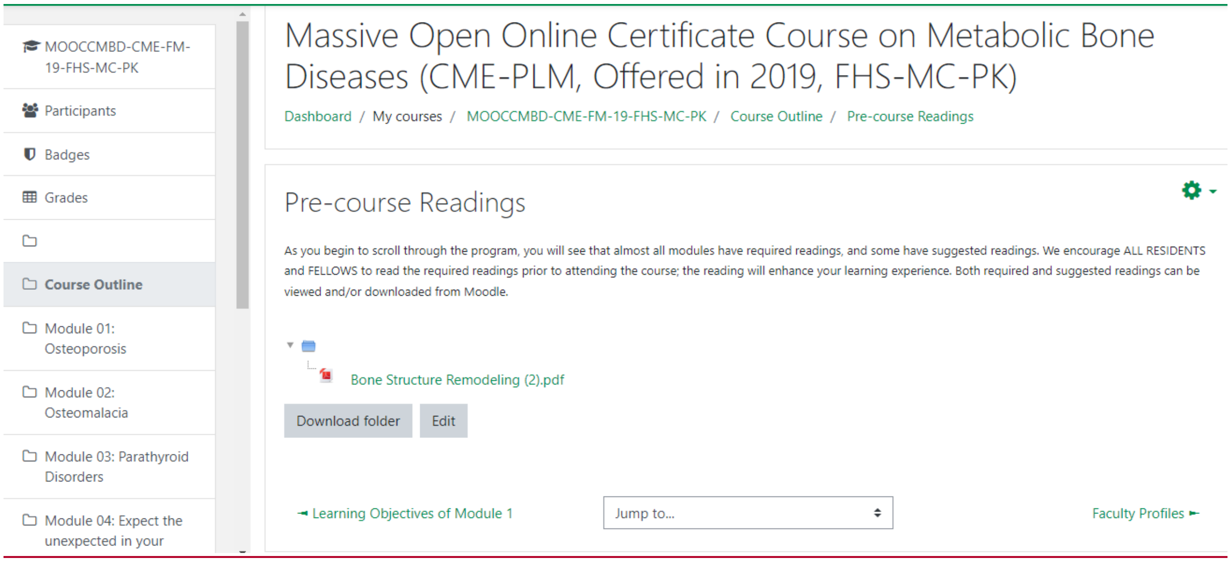
MOOCCMBD-CME-FM-19-FHS-MC-PK
Participants
Badges
Grades
Course Outline
Module 01: Osteoporosis
Module 02: Osteomalacia
Module 03: Parathyroid Disorders
Module 04: Expect the unexpected in your
Massive Open Online Certificate Course on Metabolic Bone Diseases (CME-PLM, Offered in 2019, FHS-MC-PK)
Dashboard / My courses / MOOCCMBD-CME-FM-19-FHS-MC-PK / Course Outline / Pre-course Readings
Pre-course Readings
As you begin to scroll through the program, you will see that almost all modules have required readings, and some have suggested readings. We encourage ALL RESIDENTS and FELLOWS to read the required readings prior to attending the course; the reading will enhance your learning experience. Both required and suggested readings can be viewed and/or downloaded from Moodle.
Bone Structure Remodeling (2).pdf
Download folder
Edit
Learning Objectives of Module 1
Jump to...
Faculty Profiles

## Fig 3b

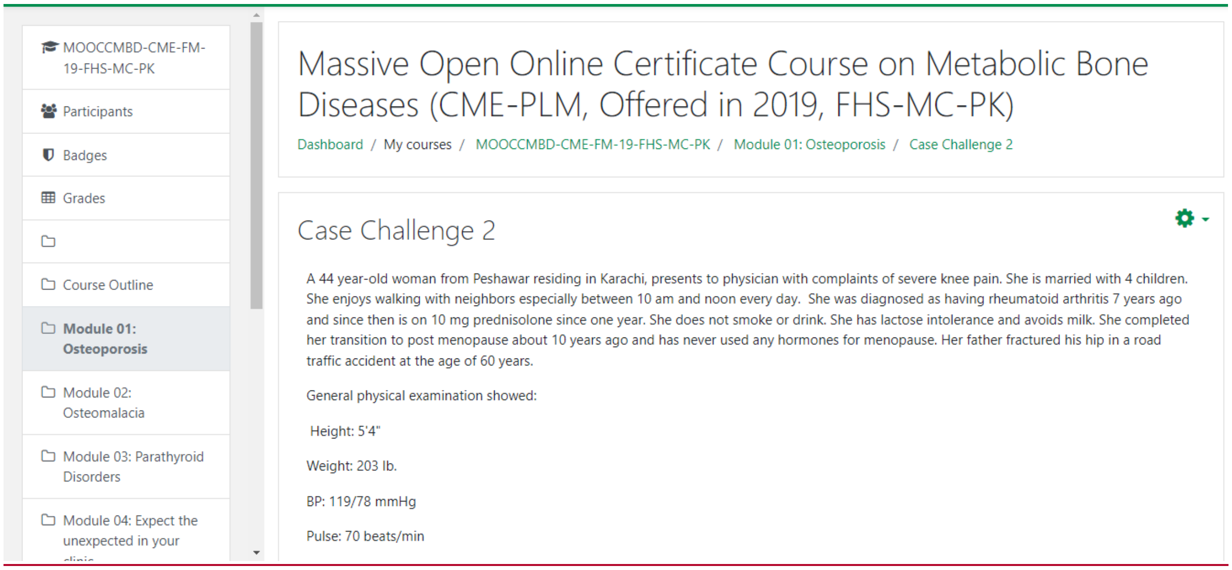
MOOCCMBD-CME-FM-19-FHS-MC-PK
Participants
Badges
Grades
Course Outline
Module 01: Osteoporosis
Module 02: Osteomalacia
Module 03: Parathyroid Disorders
Module 04: Expect the unexpected in your
Massive Open Online Certificate Course on Metabolic Bone Diseases (CME-PLM, Offered in 2019, FHS-MC-PK)
Dashboard / My courses / MOOCCMBD-CME-FM-19-FHS-MC-PK / Module 01: Osteoporosis / Case Challenge 2
Case Challenge 2
A 44 year-old woman from Peshawar residing in Karachi, presents to physician with complaints of severe knee pain. She is married with 4 children. She enjoys walking with neighbors especially between 10 am and noon every day. She was diagnosed as having rheumatoid arthritis 7 years ago and since then is on 10 mg prednisolone since one year. She does not smoke or drink. She has lactose intolerance and avoids milk. She completed her transition to post menopause about 10 years ago and has never used any hormones for menopause. Her father fractured his hip in a road traffic accident at the age of 60 years.
General physical examination showed:
Height: 5'4"
Weight: 203 lb.
BP: 119/78 mmHg
Pulse: 70 beats/min

**Fig 3c**

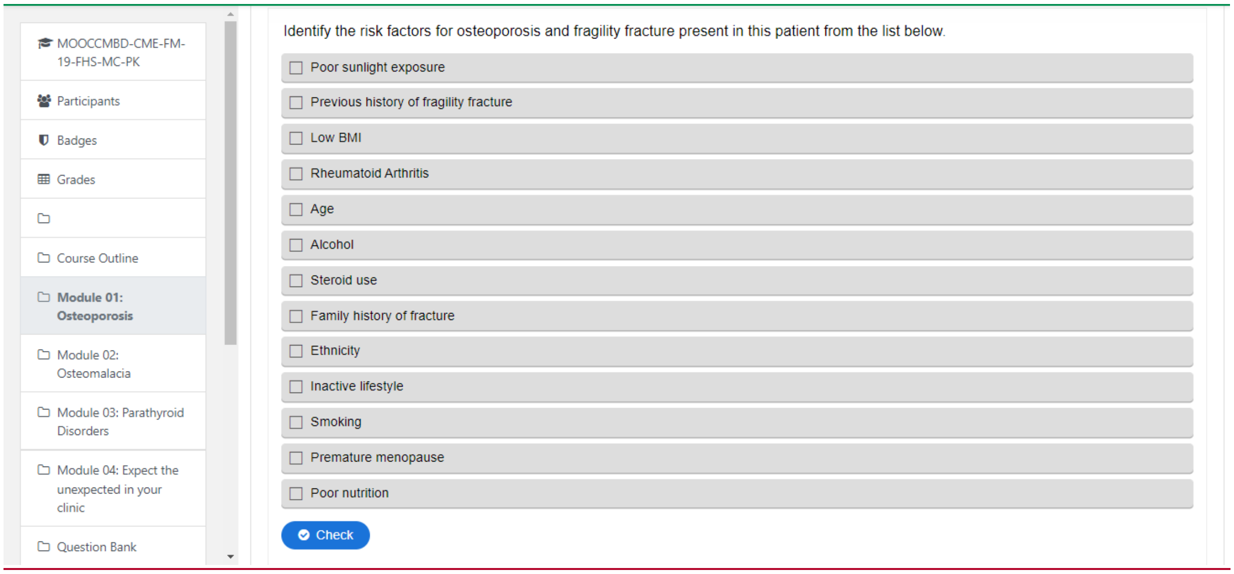
MOOCCMBD-CME-FM-19-FHS-MC-PK
Participants
Badges
Grades
Course Outline
Module 01: Osteoporosis
Module 02: Osteomalacia
Module 03: Parathyroid Disorders
Module 04: Expect the unexpected in your clinic
Question Bank
Identify the risk factors for osteoporosis and fragility fracture present in this patient from the list below.
Poor sunlight exposure
Previous history of fragility fracture
Low BMI
Rheumatoid Arthritis
Age
Alcohol
Steroid use
Family history of fracture
Ethnicity
Inactive lifestyle
Smoking
Premature menopause
Poor nutrition
Check

**Fig 4**

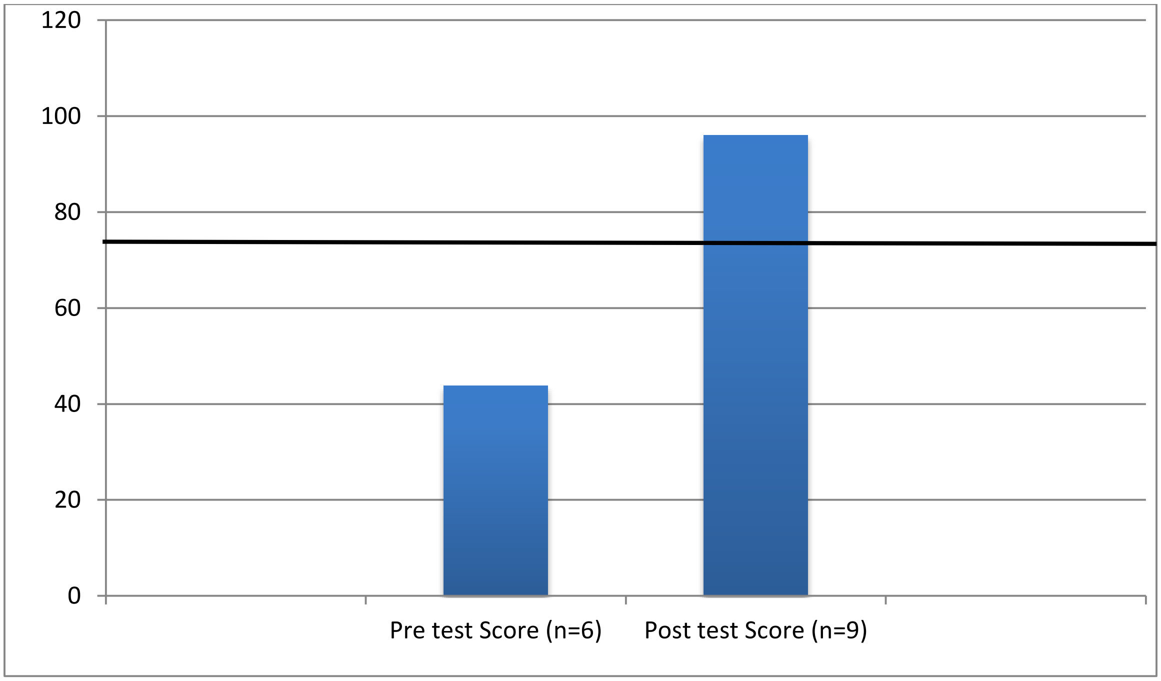
120
100
80
60
40
20
0
Pre test Score (n=6)
Post test Score (n=9)

**Fig 5a**

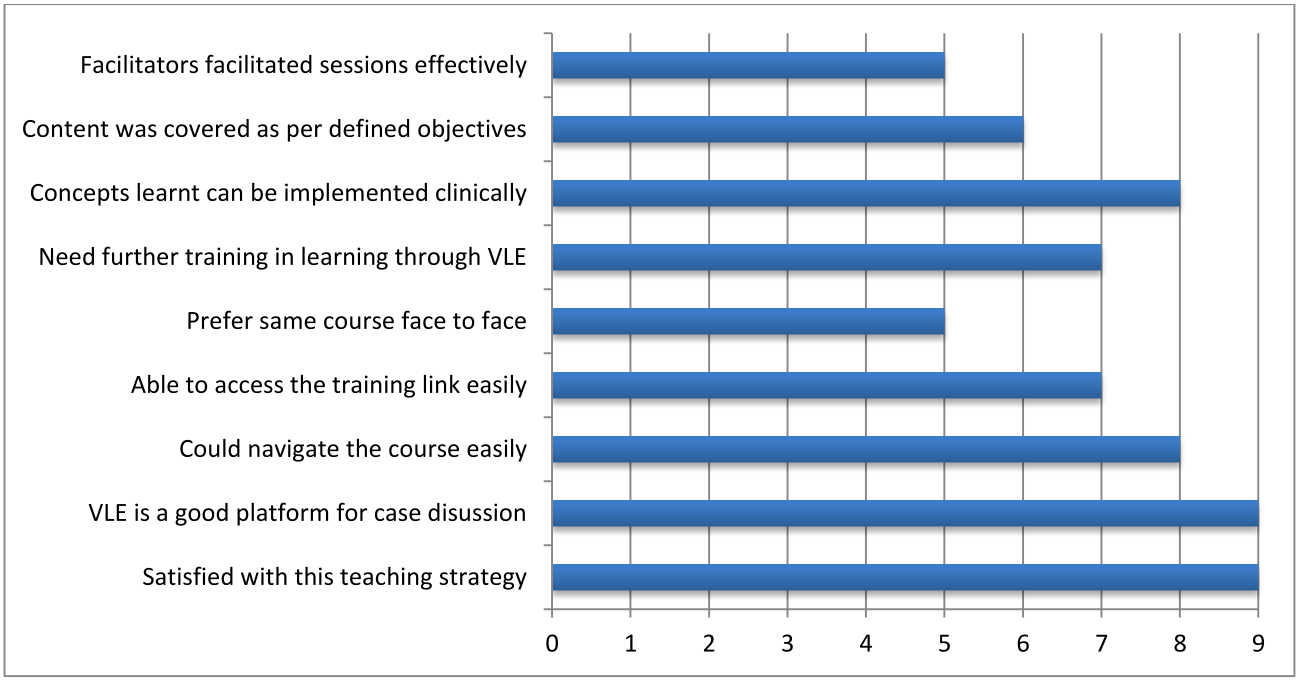

Facilitators facilitated sessions effectively
Content was covered as per defined objectives
Concepts learnt can be implemented clinically
Need further training in learning through VLE
Prefer same course face to face
Able to access the training link easily
Could navigate the course easily
VLE is a good platform for case disussion
Satisfied with this teaching strategy
0
1
2
3
4
5
6
7
8
9

**Fig 5b**

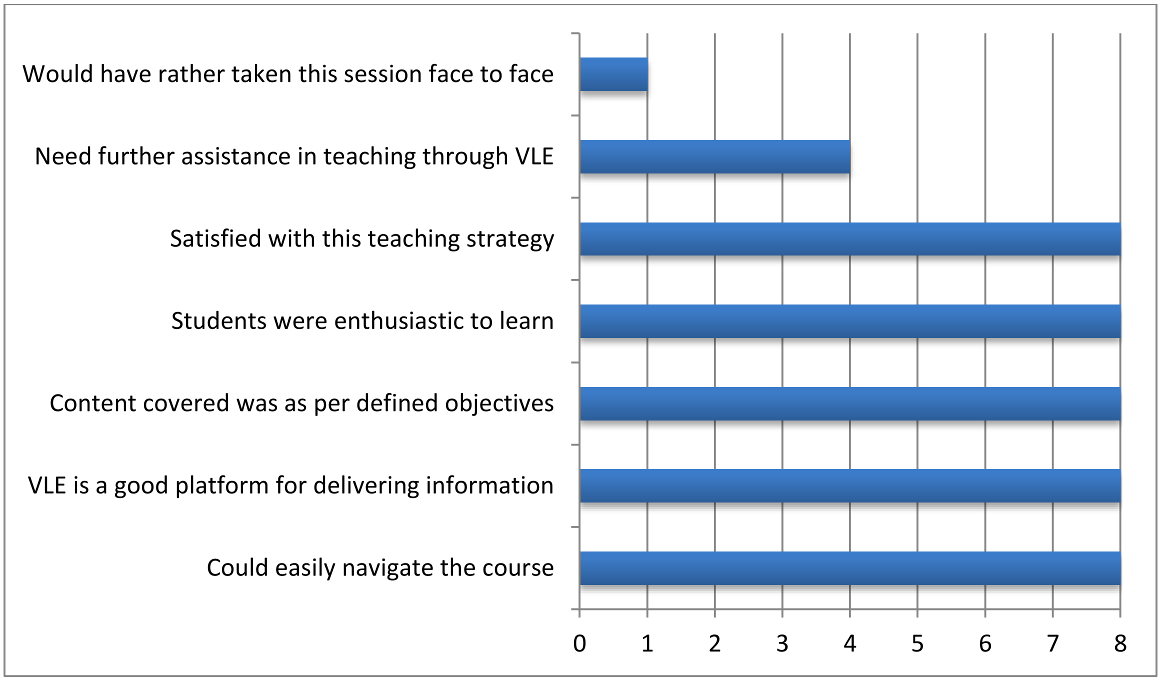

Would have rather taken this session face to face
Need further assistance in teaching through VLE
Satisfied with this teaching strategy
Students were enthusiastic to learn
Content covered was as per defined objectives
VLE is a good platform for delivering information
Could easily navigate the course
0
1
2
3
4
5
6
7
8
