# Supplementary material for: Developing and piloting an online course on osteoporosis using a multidisciplinary multi-institute approach- a cross-sectional qualitative study
Source: PLoS One. 2024 Feb 15;19(2):e0291617. doi: 10.1371/journal.pone.0291617 (PMC10868812; doi:10.1371/journal.pone.0291617)
Supplement: Attachment — Submitted filename: Review PONE-D-22-28614R1 07282023.docx [file pone.0291617.s002.docx]

Manuscript number: PONE-D-22-28614R1

Paper Review: Developing and piloting an online course on osteoporosis using a multidisciplinary multi-

institute approach- a cross-sectional qualitative study

Improvements:

1. The authors made all data underlying the findings in their manuscript fully available now.
2. I appreciate that the authors responded to my comments and revised their manuscript accordingly.

My only suggestion is that the abstract does not reflect the improvements the authors have done in the manuscript itself. Was the only goal of this study to gauge the educational impact of a novel educational tool? I don’t think that this manuscript has the sample to do this kind of evaluation in terms of educational impact. It does show the feasibility and potential of a powerful educational tool in the medical education in Pakistan. Also, the results and the conclusion in the abstract don’t seem to connect well. If the authors can summarize the strengths of qualitative part of their online course development process, the abstract will give a better overview of the approach they adopted in this manuscript.

There are a few minor points I want to address here:

1. This point may be irrelevant, and it can be ignored if the editors of this journal are fine with this. I just don’t understand why the authors responded ‘N/A’ to the Ethic Statement in their submission of this manuscript. This study did involve human participants. However, the authors did include the ethical statement in their revised manuscript. So it was taken care of in the methods section of the manuscript. Therefore, it can be an irrelevant point here.
2. Fig 5a and 5b were the count of each item in terms of course content and satisfaction of this innovative teaching and learning method? In the Phase V – course evaluation and feedback (starting from LINE 174 and ending in LINE 193 in the clean version of the revised manuscript), an online survey was on a Likert scale of 1-5. How do we interpret the fig 5a and 5b? Or are Fig 5a and 5b not related to this section?
3. In LINE 261, an extra T was put in with ‘Nine’ PG trainees in the clean version of the revised manuscript.

I recommend that this article be accepted for publication.
