# Supplementary material for: Developing and piloting an online course on osteoporosis using a multidisciplinary multi-institute approach- a cross-sectional qualitative study
Source: PLoS One. 2024 Feb 15;19(2):e0291617. doi: 10.1371/journal.pone.0291617 (PMC10868812; doi:10.1371/journal.pone.0291617)
Supplement: Attachment — Submitted filename: Rebuttal letter.docx [file pone.0291617.s003.docx]

**Authors’ Responses to Reviewers’ Comments**

**25/8/2023**

**Comments to the Author**

1. If the authors have adequately addressed your comments raised in a previous round of review and you feel that this manuscript is now acceptable for publication, you may indicate that here to bypass the “Comments to the Author” section, enter your conflict of interest statement in the “Confidential to Editor” section, and submit your "Accept" recommendation.

Reviewer #2: All comments have been addressed

Reviewer #3: (No Response)

**Authors’ Response: Thank you for your comments.**

2. Is the manuscript technically sound, and do the data support the conclusions?

The manuscript must describe a technically sound piece of scientific research with data that supports the conclusions. Experiments must have been conducted rigorously, with appropriate controls, replication, and sample sizes. The conclusions must be drawn appropriately based on the data presented.

Reviewer #2: Yes

Reviewer #3: Yes

**Authors’ Response: Thank you for your comments.**

3. Has the statistical analysis been performed appropriately and rigorously?

Reviewer #2: N/A

Reviewer #3: N/A

**Authors’ Response: Thank you for your comments.**

4. Have the authors made all data underlying the findings in their manuscript fully available?

The [PLOS Data policy](http://www.plosone.org/static/policies.action#sharing) requires authors to make all data underlying the findings described in their manuscript fully available without restriction, with rare exception (please refer to the Data Availability Statement in the manuscript PDF file). The data should be provided as part of the manuscript or its supporting information, or deposited to a public repository. For example, in addition to summary statistics, the data points behind means, medians and variance measures should be available. If there are restrictions on publicly sharing data—e.g. participant privacy or use of data from a third party—those must be specified.

Reviewer #2: Yes

Reviewer #3: Yes

**Authors’ Response: Thank you for your comments.**

5. Is the manuscript presented in an intelligible fashion and written in standard English?

PLOS ONE does not copyedit accepted manuscripts, so the language in submitted articles must be clear, correct, and unambiguous. Any typographical or grammatical errors should be corrected at revision, so please note any specific errors here.

Reviewer #2: Yes

Reviewer #3: Yes

**Authors’ Response: Thank you for your comments.**

6. Review Comments to the Author

Please use the space provided to explain your answers to the questions above. You may also include additional comments for the author, including concerns about dual publication, research ethics, or publication ethics. (Please upload your review as an attachment if it exceeds 20,000 characters)

Reviewer #2: Improvements:

1. The authors made all data underlying the findings in their manuscript fully available now.
2. I appreciate that the authors responded to my comments and revised their manuscript accordingly.

My only suggestion is that the abstract does not reflect the improvements the authors have done in the manuscript itself. Was the only goal of this study to gauge the educational impact of a novel educational tool? I don’t think that this manuscript has the sample to do this kind of evaluation in terms of educational impact. It does show the feasibility and potential of a powerful educational tool in the medical education in Pakistan. Also, the results and the conclusion in the abstract don’t seem to connect well. If the authors can summarize the strengths of qualitative part of their online course development process, the abstract will give a better overview of the approach they adopted in this manuscript.

There are a few minor points I want to address here:
1. This point may be irrelevant, and it can be ignored if the editors of this journal are fine with this. I just don’t understand why the authors responded ‘N/A’ to the Ethic Statement in their submission of this manuscript. This study did involve human participants. However, the authors did include the ethical statement in their revised manuscript. So it was taken care of in the methods section of the manuscript. Therefore, it can be an irrelevant point here.
2. Fig 5a and 5b were the count of each item in terms of course content and satisfaction of this innovative teaching and learning method? In the Phase V – course evaluation and feedback (starting from LINE 174 and ending in LINE 193 in the clean version of the revised manuscript), an online survey was on a Likert scale of 1-5. How do we interpret the fig 5a and 5b? Or are Fig 5a and 5b not related to this section?
3. In LINE 261, an extra T was put in with ‘Nine’ PG trainees in the clean version of the revised manuscript.

**Authors’ Response:**

**Thank you for your comments and suggestions! We have reworded the abstract to reflect the aims and goals of the manuscript. With regards to point 1, we have included the ethical statement in the manuscript. Point 2 stands correct- a Likert scale was not used, and the explanation has been modified to reflect that. Point 3 has also been addressed.**

Reviewer #3: (No Response)
